# *Notes from the Field:* Hospital Contact Investigation for a Patient Who Developed a Zoster Vaccine–Related Rash — Maryland, February 2015

**DOI:** 10.15585/mmwr.mm6628a6

**Published:** 2017-07-21

**Authors:** Diana P. Le, Jamie Vega, Lucas A Johnson

**Affiliations:** ^1^Uniformed Services University of the Health Sciences, Bethesda, Maryland; ^2^Walter Reed National Military Medical Center, Bethesda, Maryland.

On January 30, 2015, the public health department of a Maryland hospital was notified of a patient who developed a disseminated rash after receiving the live attenuated zoster vaccine, Zostavax (Merck). Zostavax is routinely provided to adults aged ≥50 years for the prevention of herpes zoster (shingles). The patient, a man aged 51 years, was evaluated in an outpatient clinic on postvaccination day 21, at which time physical exam revealed a nonpainful, nonpruritic, mixed maculopapular and vesicular rash (approximately 50 total lesions) involving the patient’s face, torso, groin, and arms. The patient was born and raised in Greece and reported that he did not have varicella (chickenpox) as a child, and he did not recall previous vaccination against varicella. For 10 months before developing the rash, the patient received a weekly 10-mg dose of methotrexate for rheumatoid factor-negative spondyloarthropahy. He reported no contact with persons known or suspected to have active varicella or zoster and had no cough or other constitutional symptoms. Valacyclovir, an oral antiviral medication, was prescribed, and the patient was instructed to remain at home and avoid outside contacts until the lesions crusted over. All household contacts of the patient had documented evidence of receipt of 2 doses of varicella vaccine. Direct immunofluorescence testing and culture of vesicular fluid were positive for varicella zoster virus (VZV) on postvaccination day 24.

Although zoster is less contagious than varicella (particularly if the rash is appropriately covered), the unusual presentation of the rash raised concerns for varicella. A contact investigation was initiated by the hospital’s public health department to identify health care worker contacts as well as clinic waiting room patient contacts who might have been exposed to the patient during the outpatient clinic visit. Eight health care workers were identified as having had face-to-face contact with the patient; all had documented evidence of VZV immunity by antibody titer or documentation of receipt of 2 doses of varicella vaccine. The patient spent approximately 25 minutes in the clinic waiting room, resulting in potential exposure of 18 persons. Among these persons, 15 had evidence of VZV immunity and the three who did not were offered vaccination. None of the unimmunized waiting room contacts was pregnant, had an immunocompromising medical condition, or was undergoing immunomodulatory treatment. In accordance with current guidelines, exposed health care workers were instructed to remain vigilant for signs or symptoms of varicella (i.e., fever, headache, or other constitutional symptoms and skin lesions) for 21 days after exposure and to immediately report symptoms to the hospital’s public health department if any occurred (*1*). No cases of varicella were identified in potentially exposed patients or health care workers after 21 days of surveillance. After the conclusion of the contact investigation, vesicular fluid collected and sent for genotyping at time of the initial patient evaluation demonstrated vaccine-type (Oka/Merck) virus.

Development of a generalized rash following zoster vaccination is rare, but can occur. Circulation of wild type VZV has declined considerably during the era of varicella and zoster vaccines (*2*); however, given the delays and challenges in determining if a vesicular rash in a vaccine recipient is VZV, early institution of a contact investigation by clinicians and public health officials might mitigate the risk for VZV transmission. Potentially exposed contacts without evidence of VZV immunity should be vaccinated against varicella. Both varicella vaccination and varicella zoster immune globulin are most effective in preventing disease in susceptible persons when administered as soon as possible after exposure (*3*).

Health care settings should ensure that health care workers are immune to VZV. Documentation of immunity includes: 1) written documentation of vaccination with 2 doses of varicella vaccine; 2) laboratory evidence of immunity or laboratory confirmation of disease; 3) diagnosis or verification of a history of varicella disease by a health care provider; or 4) diagnosis or verification of a history of herpes zoster by a health care provider (*4*,*5*). This case highlights the importance of maintaining vigilance for unusual events following the use of live vaccines in persons who receive immunosuppressant medications, the importance of vaccination for primary prevention of communicable diseases in hospital settings, and the value of a robust occupational health program as a critical component of hospital infection control efforts.

## References

[1] CDC. Preventing varicella-zoster virus (VZV) transmission from zoster in healthcare settings. Atlanta, GA: US Department of Health and Human Services, CDC; 2014. https://www.cdc.gov/shingles/hcp/HC-settings.html#healthcare-personnel

[2] Lopez AS, Zhang J, Marin M. Epidemiology of varicella during the 2-dose varicella vaccination program—United States, 2005–2014. MMWR Morb Mortal Wkly Rep 2016;65:902–5. 27584717 10.15585/mmwr.mm6534a4

[3] Lopez AS, Marin M. Strategies for the control and investigation of varicella outbreaks. Atlanta, GA: US Department of Health and Human Services, CDC; 2008. https://www.cdc.gov/chickenpox/outbreaks/downloads/manual.pdf

[4] Marin M, Güris D, Chaves SS, Schmid S, Seward JF; Advisory Committee on Immunization Practices, CDC. Prevention of varicella: recommendations of the Advisory Committee on Immunization Practices (ACIP). MMWR Recomm Rep 2007;56(No. RR-4). 17585291

[5] Shefer A, Atkinson W, Friedman C, ; Advisory Committee on Immunization Practices, CDC. Immunization of health-care personnel: recommendations of the Advisory Committee on Immunization Practices (ACIP). MMWR Recomm Rep 2011;60(No. RR-7). 22108587

